# Atherogenic Cytokines Regulate VEGF-A-Induced Differentiation of Bone Marrow-Derived Mesenchymal Stem Cells into Endothelial Cells

**DOI:** 10.1155/2015/498328

**Published:** 2015-05-28

**Authors:** Izuagie Attairu Ikhapoh, Christopher J. Pelham, Devendra K. Agrawal

**Affiliations:** ^1^Department of Medical Microbiology and Immunology, Creighton University School of Medicine, Omaha, NE 68178, USA; ^2^Department of Biomedical Sciences, Creighton University School of Medicine, Omaha, NE 68178, USA; ^3^Center for Clinical and Translational Science, Creighton University School of Medicine, Omaha, NE 68178, USA

## Abstract

Coronary artery stenting or angioplasty procedures frequently result in long-term endothelial dysfunction or loss and complications including arterial thrombosis and myocardial infarction. Stem cell-based therapies have been proposed to support endothelial regeneration. Mesenchymal stem cells (MSCs) differentiate into endothelial cells (ECs) in the presence of VEGF-A* in vitro*. Application of VEGF-A and MSC-derived ECs at the interventional site is a complex clinical challenge. In this study, we examined the effect of atherogenic cytokines (IL-6, TNF*α*, and Ang II) on EC differentiation and function. MSCs (CD44^+^, CD73^+^, CD90^+^, CD14^−^, and CD45^−^) were isolated from the bone marrow of Yucatan microswine. Naïve MSCs cultured in differentiation media containing VEGF-A (50 ng/mL) demonstrated increased expression of EC-specific markers (vWF, PECAM-1, and VE-cadherin), VEGFR-2 and Sox18, and enhanced endothelial tube formation. IL-6 or TNF*α* caused a dose-dependent attenuation of EC marker expression in VEGF-A-stimulated MSCs. In contrast, Ang II enhanced EC marker expression in VEGF-A-stimulated MSCs. Addition of Ang II to VEGF-A and IL-6 or TNF*α* was sufficient to rescue the EC phenotype. Thus, Ang II promotes but IL-6 and TNF*α* inhibit VEGF-A-induced differentiation of MSCs into ECs. These findings have important clinical implications for therapies intended to increase cardiac vascularity and reendothelialize coronary arteries following intervention.

## 1. Introduction

Cardiovascular diseases are the foremost cause of mortality in the United States [1]. Myocardial infarction often results from atherosclerotic occlusion of coronary arteries. Interventional procedures, including angioplasty and stenting, are performed in order to restore cardiac blood flow and function [2]. At the site of intervention, denudation and dysfunction of endothelial cells (ECs) contribute to thrombosis, intimal hyperplasia, and restenosis [2].

Recent studies have demonstrated the importance of circulating progenitor cells in maintaining normal endothelial function as well as endothelial repair after vascular injury [3]. MSCs are a potential source of autologous ECs [4–6]. MSCs are multipotent cells capable of differentiating into cells of mesodermal lineage. Upon vascular injury, the endothelium engages in* de novo* synthesis of cytokines, chemokines, and growth factors, including VEGF-A [7]. VEGF-A promotes EC proliferation and angiogenesis. VEGF-A also acts as a mitogen to attract peripheral stem cells [7–9]. VEGF-A orchestrates the differentiation of bone marrow-derived MSCs (BM-MSCs) into ECs* in vitro *[5, 9–11]. The emergent ECs can then be transplanted into the site of occlusion or into the ischemic myocardium. However, the local site of occlusive arterial disease consists of a complex inflammatory cytokine milieu [12].

Atherogenic cytokines play important roles in the inflammatory response at the site of plaque formation, and their synthesis and secretion are dynamically regulated. In particular, there is little to no basal expression of IL-6 and TNF*α* by healthy endothelium [13–15]. Likewise, IL-6 receptors and TNF*α* receptors are not basally expressed on BM-MSCs [4, 6]. The levels of IL-6, TNF*α* and Ang II have been shown to increase in arteries in response to intervention [15–17]. Therefore, it is important to consider the effects of these factors on MSCs intended for transplantation. The goal of the present study was to investigate the effect of key atherogenic cytokines on EC differentiation and function.

## 2. Materials and Methods

### 2.1. Swine Model

All animal procedures were in compliance with applicable federal, state, and local laws and regulations and institutional policies. Animal work was performed in accordance with the guidelines set by the National Institutes of Health for the care and use of experimental animals. The Creighton University Institutional Animal Care and Use Committee approved the animal research protocol. The swine model of coronary artery intimal hyperplasia was implemented, as previously established by our group [18, 19]. Yucatan microswine (30–40 lbs) was purchased from Sinclair Bioresources (Windham, Maine). Microswine were maintained on high cholesterol diet (Harlan Laboratories). After 6 months, microswine in the interventional group were subjected to percutaneous transluminal balloon angioplasty (PTCA) in the left circumflex artery (LCX). The animals were sacrificed 4 months after angioplasty. Age-matched control microswine were maintained on high cholesterol diet and received no PTCA interventional procedures.

### 2.2. MSC Isolation and Differentiation

For cell culture studies, MSCs were isolated from femur bone marrow of Yucatan microswine as previously reported by our group [20, 21]. MSCs used for experiments in this study were between passages 3 and 5. The isolated naïve MSCs were characterized as highly immunopositive for the expression of stem cell markers, CD44, CD73, and CD90, as determined by flow cytometry. The cells from the same gate were negative for the macrophage marker, CD14, and the hematopoietic stem cell marker, CD45. Growth media consisting of Dulbecco modified eagle medium (DMEM) with 10% fetal bovine serum (FBS) were used to harvest and culture MSCs. EC differentiation media (DM) was endothelial growth media 2 (EGM-2) containing 50 ng/mL of recombinant human VEGF-A_165_ (Peprotech, Rocky Hill, New Jersey). In further experiments, naïve MSC cultures were differentiated in the presence of 1–100 pg/mL of IL-6 (Peprotech, Rocky Hill, New Jersey) and/or TNF*α* (Peprotech, Rocky Hill, New Jersey) and/or 2–50 ng/mL Ang II (Sigma). For experiments designed for costimulation with cytokines, MSCs were pretreated with Ang II for 1 hr, and then VEGF-A plus IL-6 and/or TNF*α* were added to the culture. For cotreatment with Ang II and VEGF-A together, the peptides were added at the same time. For cotreatment with IL-6 and/or TNF*α* and VEGF-A together, the agents were added at the same time. Basic EGM-2 was the negative control for DM. Stimulation of differentiation was initiated when naive MSCs were 50% confluent and the protocol continued for 10 days. The cell cultures were maintained at 37°C in a humidified atmosphere containing 5% CO_2_. Media containing various combinations of VEGF-A, Ang II, and IL-6 and TNF*α* was changed every 48 hrs.

### 2.3. Multilineage Differentiation Potential of MSCs

Mesenchymal capacity of bone marrow cells was proved by trilineage differentiation into osteogenic, chondrogenic, and adipogenic lineages. In brief, during osteogenic and adipogenic differentiation, the cells were cultured in a 6-well plate and induction medium was added at 50% confluency. For osteogenesis, the cells were analyzed by alizarin red stain after 14 days and adipogenesis differentiation by oil red O staining after 21 days of stimulation. To test for chondrogenic differentiation, the cells were cultured in induction media for 21 days and then stained with Alcian blue/Safranin O. Reagents were from Sigma Aldrich Company.

### 2.4. FACS Characterization of Naïve MSCs and ECs

Flow cytometry was performed using standard methods and carried out on a BD FACSAria I/II System (BD Biosciences, San Jose, CA). First, cells (~1 × 10^6^/mL) were washed with PBS containing 4% FBS and incubated with primary antibodies conjugated to fluorophore (FITC) for 30 min at 4°C in the dark. The antibody concentrations followed the specifications of the manufacturer. After 3 washes in PBS, cells were resuspended in FACS-FIX. MSCs were characterized as CD14^−^CD45^−^CD44^+^CD73^+^CD90^+^. At the end of the 10-day differentiation protocol, MSCs were analyzed for the EC markers PECAM-1-APC (ebiosciences, San Diego, CA), VE-cadherin-FITC (ebiosciences, San Diego, CA), and vWF-PE (R&D Systems, Minneapolis, MN).

### 2.5. Western Blot

Total protein lysates were isolated and quantified by Bradford assay. The lysates were separated by 10% SDS-PAGE and transferred onto a nitrocellulose membrane (BioRad, Hercules, CA). The membrane was incubated in blocking solution (1x TBS, pH 7.6, 0.1% Tween-20, and 5% w/v of nonfat dry milk) and then incubated with a primary antibody to detect Sox18 (Abcam ab23342). The membrane was probed for GAPDH (NOVUS Biological, NB300-221) to normalize the protein loading. The membrane was then incubated with HRP-conjugated secondary antibody (1 : 1000) in blocking solution for 1 hour at room temperature. HRP activity was detected by incubating the membrane in chemiluminescence solution (Bio-Rad, Hercules, CA). The exposure time was adjusted to keep the integrated optical densities within a linear and nonsaturated range. Densitometric analysis was done using a UVP Bioimaging system (UVP, Minneapolis, MN).

### 2.6. Quantitative RT-PCR

Using Trizol reagent protocol (Sigma), total RNA was isolated from naïve MSCs according to the manufacturer's instructions. The quality and quantity of RNA were quantified using a Nanodrop (Thermo-Scientific, Rockford, IL). First-strand cDNA synthesis was performed following the manufacturer's instructions (Improm II reverse transcription kit; Promega, Madison, WI) using oligo dT primers. Real-time qPCR was performed in triplicate using SYBR Green Master Mix and a Real-time PCR system (CFX96; BioRad Laboratories, Hercules, CA). The following primers were used:

 AT2R-F: 5′-GTTCCCCTTGTTTGGTGTAT-3′.
 AT2R-R: 5′-CATCTTCAGGACTTGGTCAC-3′.
 GAPDH-F: 5′-CCCATCACCATCTTCCAGGAG-3′.
 GAPDH-R: 5′-GTTGTCATGGATGACCTTGGCC-3′.
 IL-6-R-F: 5′-GCCGTGTTACTGGTGAGGAA-3′.
 IL-6-R-R: 5′-AACTGGCAGAAAAACCGCTGC-3′.
 TNF*α*-R-F 5′-CCCGAGTCTCAACCCTCAAC-3′.
 TNF*α*-R-R 5′-GTTCCTTCAAGCTCCCCCTC-3′.
 VEGFR-2-F 5′-CTGGATTCGTGGAGGAGAAATC-3′.
 VEGFR-2-R 5′-GAGATGCTCCAAGGTCAGAAAG-3′.

### 2.7. Angiogenesis Assay

Following 10 days of stimulation with VEGF or cytokine/hormone supplemented DM, MSCs were harvested and an angiogenesis assay was performed according to manufacturer's protocol (Chemicon, Temecula, CA). Polymerized EC background was prepared by incubating 100 *µ*L ECMatrix gel solution into each well of a 24-well plate at 37°C for 1 h. The stimulated cells were seeded at a concentration of 1 × 10^4^ cells on EC matrices. EGM-2 medium (300 *µ*L) was added to each well and maintained at 37°C and 5% CO_2_ incubator for 6 hrs. The formation of capillary tubes was analyzed using an inverted phase contrast microscope (Model CKK41, Olympus, Tokyo, Japan).

### 2.8. Immunofluorescence

Blocking solution against nonspecific binding contained phosphate-buffered saline (PBS), 0.25% Triton X-100, 10 mg/mL bovine serum albumin (BSA), and 5% normal goat serum (Jackson Laboratories, West Grove, PA) and was used for 1 hour at room temperature. The cells were then incubated with primary antibodies selective for anti-Ang II (Abcam ab47831), anti-IL-6 (Abcam ab6672), and anti-TNF*α* (Abcam ab2271) for 1 hour at room temperature. After washing with PBS containing 0.1% BSA three times for 5 min each, a secondary antibody (affinity purified goat anti-rabbit Cy2 & Cy3 antibody, 1 : 500) was applied to the sections for 1 hour in the dark to visualize immunofluorescent cells (Jackson Immunolabs, West Grove, PA). Negative controls were run in parallel either by using rabbit preimmune serum PAC-767 (Pacific Immunology, Ramona, CA) instead of primary antibody or by complete omission of primary antibody. Negative control was absent of staining. Sections were washed with PBS with 0.1% BSA three times for 5 min and dipped into distilled water for 2 sec. Fluorescence was preserved by sealing specimens with a solution of equal parts of PBS and glycerol containing 10 mg/mL n-propyl gallate and 1.5 mg/mL 4′,6-diamidino-2-phenylindole (DAPI). To prevent the escape of the mounting medium from the coverslips, a single layer of nail polish was placed around the edges. Pictures were taken within 1 hour of the mounting using an Olympus DP71 camera (Olympus, St Louis, MO).

### 2.9. Statistical Analysis

Data are presented as the mean ± standard deviation (SD). For each experiment, stem cells were isolated from the femoral bone of separate pigs. Data were analyzed using GraphPad Prism. Multiple group comparisons were performed by Bonferroni's multiple comparison test using One-way ANOVA. Probability (*p*) value < 0.05 was accepted as statistically significant.

## 3. Results

### 3.1. Characterization of BM-MSCs

**Figure 1:**
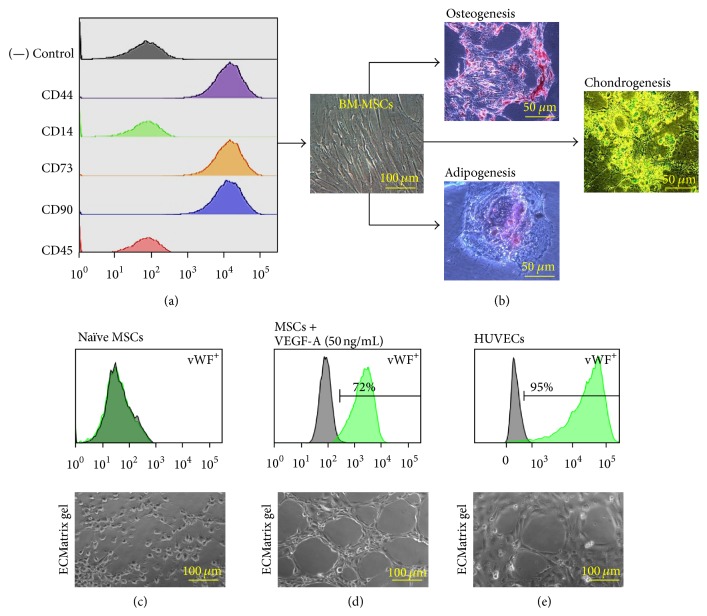
Characterization of BM-MSCs: detailed FACS characterization revealed that MSCs at P3 to P5 stained negatively for CD14 and CD45 but expressed surface markers that are indicative of MSC lineage, including CD44, CD73, and CD90 (a). Isolated MSCs also exhibited fibroblastoid-like morphology (b). Naïve BM-MSCs demonstrated the capacity to differentiate in osteogenic, chondrogenic, and adipogenic lineages. Alizarin red showed staining of calcium deposits in MSCs differentiated into the osteogenic lineage. Alcian/Safranin O blue showed staining of peptidoglycans characteristic of differentiation into the chondrogenic lineage. Oil Red O showed staining of the lipids and triglycerides, indicating differentiation into the adipogenic lineage. Each image shown is representative of independent experiments performed with BM-MSC cultures derived from separate microswine (*n* = 3). FACS analysis performed for EC markers on naïve MSCs (c), MSCs treated with VEGF-A (d), and HUVECs (e). Each grid shown is representative of independent experiments performed with cultures derived from bone marrow of separate microswine (*n* = 3–6).

Primary cultures of freshly isolated MSCs from swine bone marrow were established from the adherent cells. FACS analysis revealed that the isolated MSCs were negative for CD14 and CD45 and immunopositive for CD44 (hyaluronic acid receptor), CD73 (5′-nucleotidase), and CD90 (Thy-1) (Figure 1(a)). MSCs displayed the typical fibroblastoid morphology (Figure 1(b)). Furthermore, multilineage differentiation of the immunophenotyped MSCs into adipocytes, osteocytes, and chondrocytes confirmed their stem-like nature (Figure 1(b)). Naïve MSCs did not express EC markers (Figure 1(c)). When subjected to an angiogenesis assay, naïve MSCs failed to display capillary tube formation, which is a characteristic property of ECs (Figure 1(c)). Differentiation of MSCs for 10 days in DM containing VEGF-A (50 ng/mL) increased the percentage of cells immunopositive for the expression of EC markers to >70% of the total gated cells (Figure 1(d)). The differentiated ECs displayed functional capillary tube formation in the angiogenesis assay (Figure 1(d)). HUVECs were used as positive control for the EC phenotype. HUVECs were ≥90% immunopositive for the EC markers (Figure 1(e)). HUVECs also displayed robust capillary tube formation (Figure 1(e)).

### 3.2. Balloon Angioplasty Induces Expression of IL-6, TNF*α*, and Ang II

**Figure 2:**
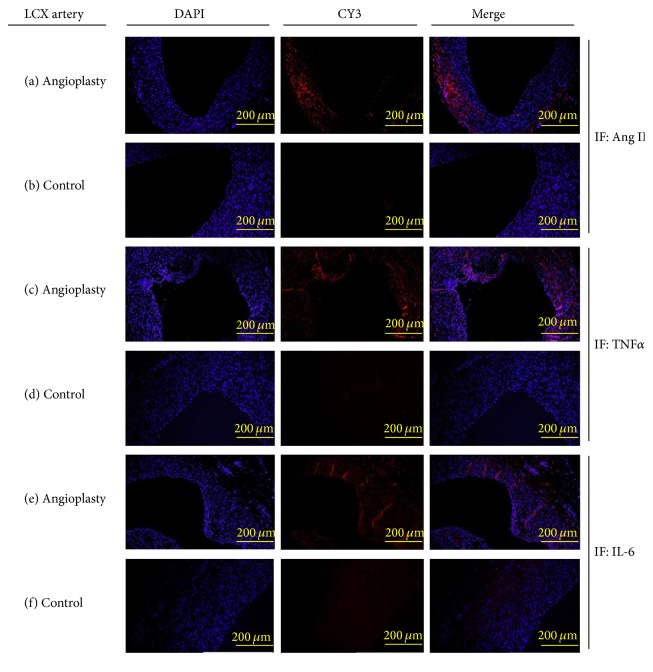
Angioplasty increases the local production of atherogenic cytokines: histological evaluation was performed on LCX arteries taken from hypercholesterolemic microswine at 4 months after angioplasty and age-matched control hypercholesterolemic microswine. The immunostaining was assessed for Ang II ((a)-(b)), TNF*α* ((c)-(d)), and IL-6 ((e)-(f)). Each image shown is representative of independent experiments performed from separate microswine (*n* = 3).

We used microswine that were maintained on high cholesterol diet and underwent PTCA intervention in the LCX artery. The animals were sacrificed 4 months after angioplasty, allowing time for coronary artery intimal hyperplasia to develop, as previously described by our group [18, 19]. Age-matched control microswine were maintained on high cholesterol diet and received no angioplasty. The degree of hypercholesterolemia, as measured by serum total cholesterol levels, was similar between the PTCA group (446 ± 70 mg/dL) and the control group (473 ± 61 mg/dL). Immunofluorescence was performed on LCX arteries in order to characterize the local cytokine/hormone milieu at the arterial intervention site. LCX arteries that underwent angioplasty were immunopositive for Ang II (Figure 2(a)), TNF*α* (Figure 2(c)), and IL-6 (Figure 2(e)). The immunostaining of these molecules was prominent in the medial and neointimal layers of LCX arteries after angioplasty. Noninjured control arteries displayed little to no basal levels of Ang II (Figure 2(b)), TNF*α* (Figure 2(d)), or IL-6 (Figure 2(f)). The results indicate that IL-6, TNF*α*, and Ang II levels are increased in coronary arteries as a result of arterial injury following PTCA interventional procedures. These inflammatory mediators could have detrimental effects against arterial repair and endothelial function.

### 3.3. IL-6 and TNF*α* Are Negative Regulators of EC Phenotype

**Figure 3:**
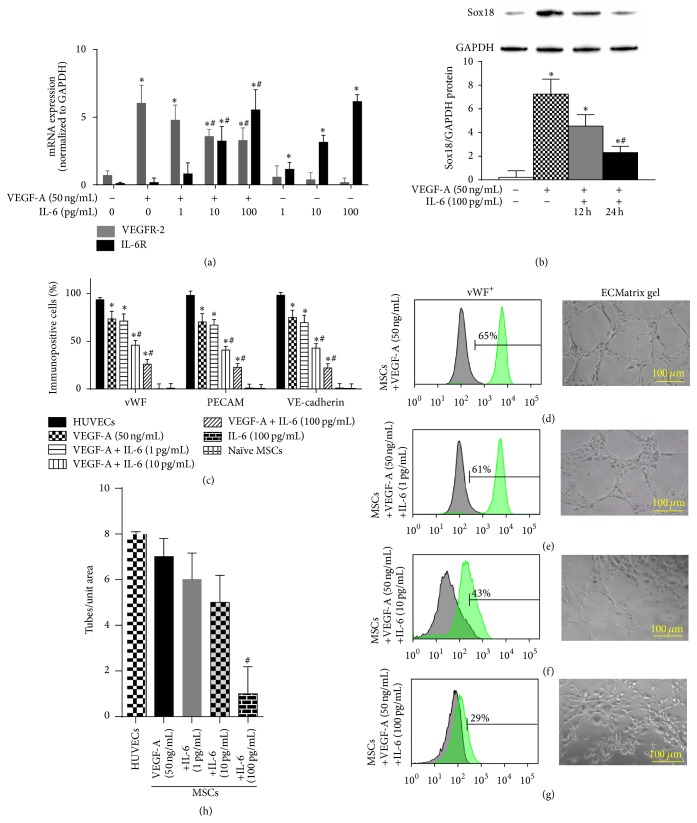
IL-6 negatively regulates EC differentiation: the effect of IL-6 on VEGF-A-stimulated differentiation of MSCs into ECs was examined. IL-6R and VEGFR-2 mRNA expression was analyzed by RT-PCR and normalized to GAPDH (*n* = 3) (a). Sox18 protein levels were measured by Western blot analysis and normalized to GAPDH (*n* = 3) (b). Expression of EC markers was determined by FACS analysis, and a representative grid is shown (*n* = 3–6) ((c)–(g)). Endothelial tube formation was examined using an angiogenesis assay (*n* = 3) ((d)–(h)). Experiments were performed with samples taken from independent BM-MSC cultures from separate microswine. HUVECs were excluded from statistical analyses. Data are shown as mean ± SD. ^*∗*^ *p* < 0.05 versus naïve MSCs and ^#^ *p* < 0.05 MSCs treated with VEGF-A versus VEGF-A plus IL-6 cotreatment.

Mechanistically, VEGF-A alone induced the mRNA expression of its receptor VEGFR-2 (Figure 3(a)) and the protein expression of the transcription factor Sox18 (Figure 3(b)). Our group recently demonstrated that Sox18 mediates VEGF-A-induced differentiation of bone marrow-derived MSCs into ECs [21]. In tandem with VEGF-A, IL-6 caused a dose-dependent increase in IL-6R mRNA and a dose-dependent decrease in VEGFR-2 mRNA (Figure 3(a)) and Sox18 protein (Figure 3(b)). Differentiation of bone marrow-derived MSCs with VEGF-A (50 ng/mL) induced the expression of EC markers (Figure 1). IL-6 cotreatment inhibited VEGF-A-mediated induction of EC marker expression (Figures 3(c), 3(d)–3(g)). IL-6 also caused a functional decrease in capillary tube formation (Figures 3(d)–3(h)).

**Figure 4:**
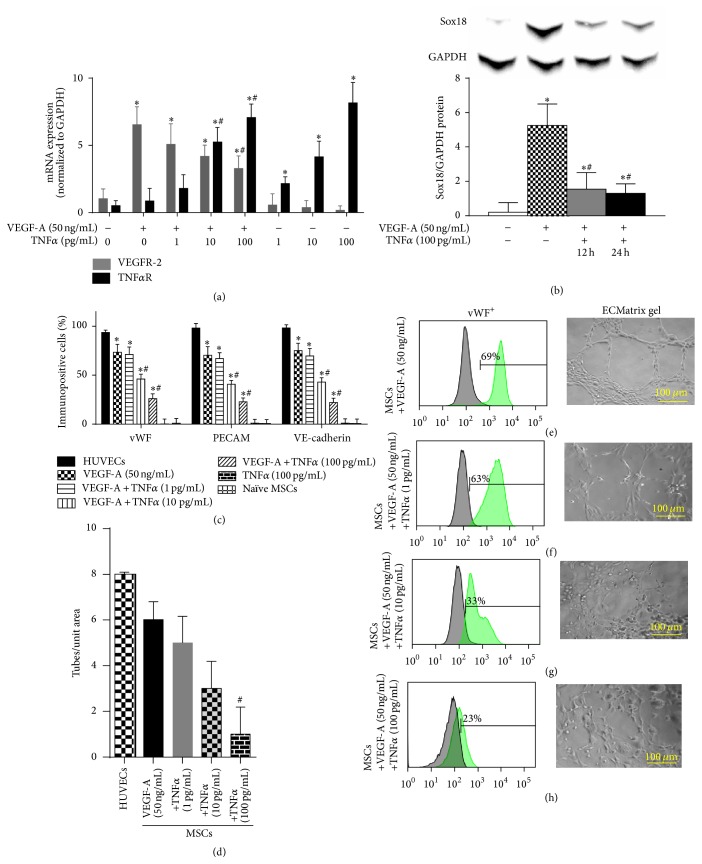
TNF*α* negatively regulates EC differentiation: the effect of TNF*α* on VEGF-A-stimulated differentiation of MSCs into ECs was examined. TNF*α*R and VEGFR-2 mRNA expression was analyzed by RT-PCR (*n* = 3) (a). Sox18 protein levels were measured by Western blot analysis (*n* = 3) (b). Expression of EC markers was determined by FACS analysis, and a representative grid is shown (*n* = 3-4) ((c)–(g)). Endothelial tube formation was examined using an angiogenesis assay (*n* = 3) ((d)–(h)). Experiments were performed with samples taken from independent BM-MSC cultures from separate microswine. HUVECs were excluded from statistical analyses. Data are shown as mean ± SD. ^*∗*^ *p* < 0.05 versus naïve MSCs and ^#^ *p* < 0.05 MSCs treated with VEGF-A versus VEGF-A plus TNF*α* cotreatment.

Similar to IL-6, TNF*α* also caused a dose-dependent increase in TNF*α*-R mRNA and a dose-dependent decrease in VEGFR-2 mRNA (Figure 4(a)) and Sox18 protein (Figure 4(b)). TNF*α* cotreatment inhibited VEGF-A-mediated induction of EC marker expression (Figures 4(c), 4(d)–4(g)) and inhibited angiogenesis (Figures 4(d)–4(h)). Thus, IL-6 and TNF*α* are negative regulators of the EC phenotype and MSC differentiation into ECs.

### 3.4. Ang II Is a Positive Regulator of EC Phenotype

**Figure 5:**
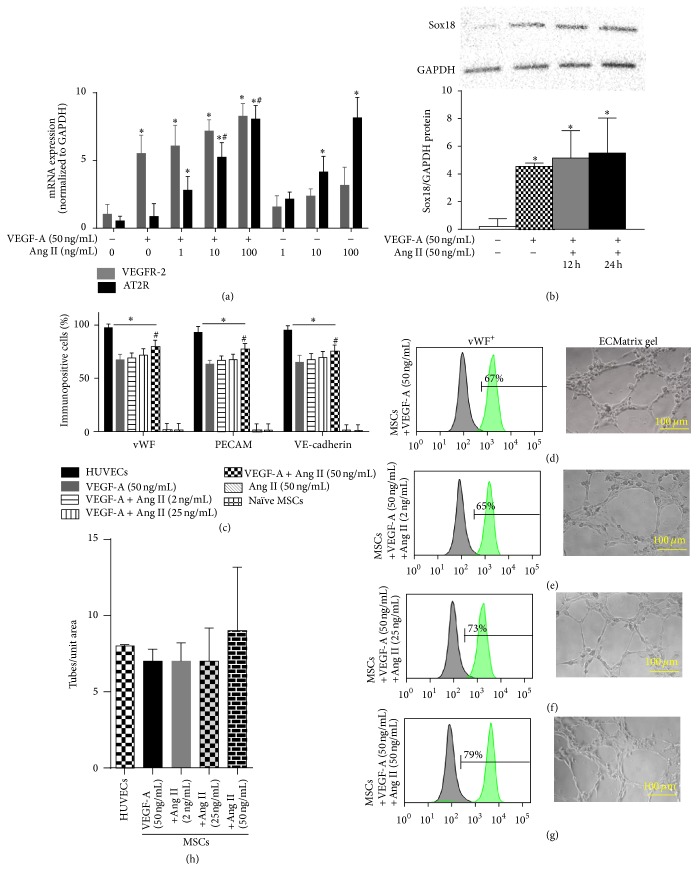
Ang II positively regulates VEGF-A-mediated EC differentiation: the effect of Ang II on VEGF-A-stimulated differentiation of MSCs into ECs was examined. AT2R and VEGFR-2 mRNA expression was analyzed by RT-PCR (*n* = 3) (a). Sox18 protein levels were measured by Western blot analysis (*n* = 3) (b). Expression of EC markers was determined by FACS analysis, and a representative grid is shown (*n* = 5-6) ((c)–(g)). Endothelial tube formation was examined using an angiogenesis assay (*n* = 3) ((d)–(h)). Experiments were performed with samples taken from independent BM-MSC cultures from separate microswine. HUVECs were excluded from statistical analyses. Data are shown as mean ± SD. ^*∗*^ *p* < 0.05 versus naïve MSCs and ^#^ *p* < 0.05 MSCs treated with VEGF-A versus VEGF-A plus Ang II cotreatment.

Ang II is also known to act as a proinflammatory mediator. In contrast to IL-6 or TNF*α*, cotreatment of MSCs with Ang II with VEGF-A increased the percentage of cells immunopositive for vWF, PECAM-1, and VE-cadherin, compared to VEGF-A alone (Figures 5(c), 5(d)–5(g)). MSCs differentiated with Ang II and VEGF-A retained the ability to form capillary tubes (Figures 5(d)–5(h)). Ang II cotreatment with VEGF-A further upregulated expression of VEGFR-2 and AT2R mRNA (Figure 5(a)) and Sox18 protein (Figure 5(b)), compared to VEGF-A alone. Unlike IL-6 and TNF*α*, Ang II is a positive regulator of the EC phenotype and MSC differentiation into ECs.

### 3.5. Ang II Opposes IL-6- or TNF*α*-Mediated Inhibition of VEGF-A-Stimulated Differentiation of MSCs into ECs

**Figure 6:**
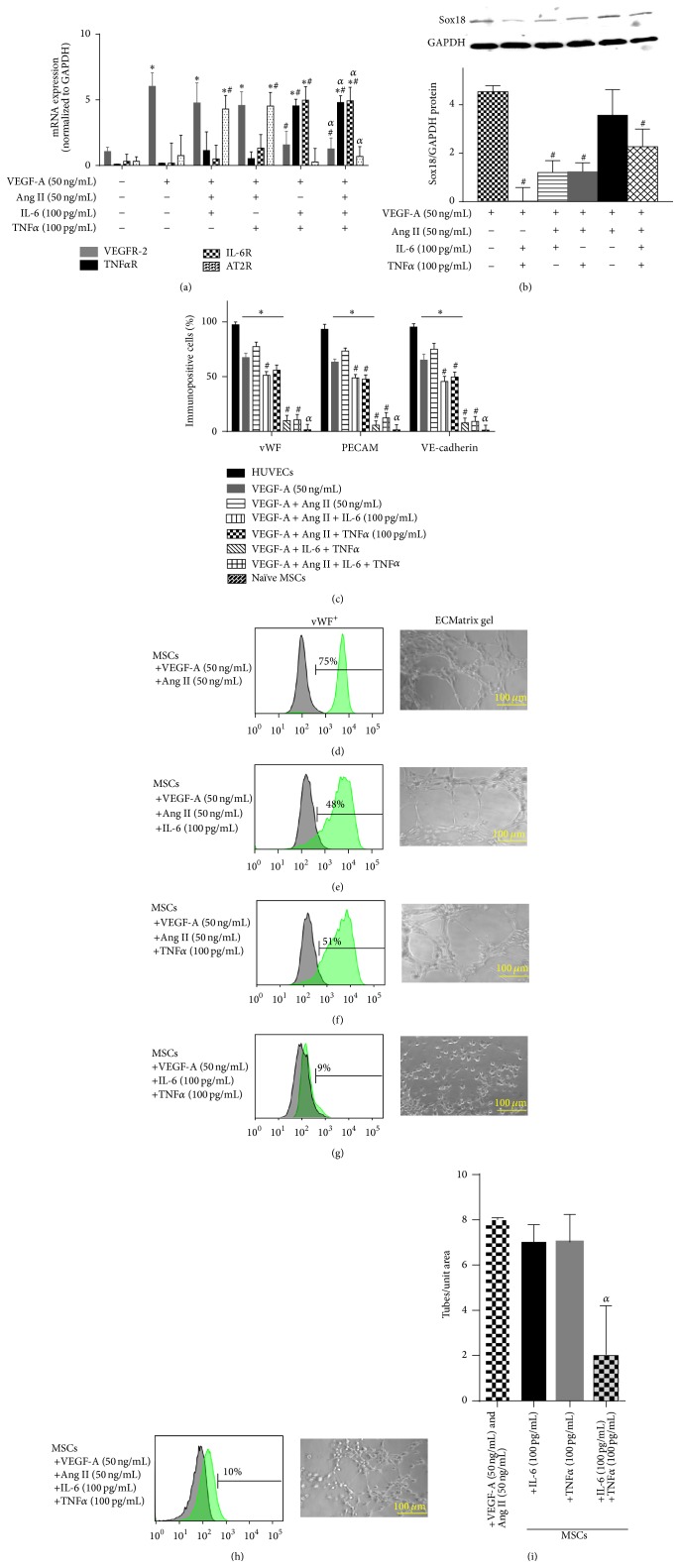
Ang II counteracts IL-6 or TNF*α* inhibition of EC differentiation: the combined effects of Ang II, IL-6, and TNF*α* on VEGF-A-stimulated differentiation of MSCs into ECs were examined. TNF*α*R, IL-6R, AT2R, and VEGFR-2 mRNA expression was analyzed by RT-PCR (*n* = 3) (a). Sox18 protein levels were measured by Western blot analysis (*n* = 3) (b). Expression of EC markers was determined by FACS analysis, and a representative grid is shown (*n* = 3-4) ((c)–(h)). Endothelial tube formation was examined using an angiogenesis assay (*n* = 3) ((d)–(i)). Experiments were performed with samples taken from independent BM-MSC cultures from separate microswine. HUVECs were excluded from statistical analyses. Data are shown as mean ± SD. ^*∗*^ *p* < 0.05 versus naïve MSCs, ^#^ *p* < 0.05 MSCs treated with VEGF-A versus VEGF-A plus Ang II cotreatment, and ^*α*^ *p* < 0.05 versus MSCs treated with VEGF-A and Ang II versus VEGF-A and Ang II plus IL-6 and/or TNF*α*.

Next we tested the effects of combinations of cytokines on the differentiation of MSCs into ECs. Treatment of naïve MSCs with the combination of IL-6, VEGF-A, and Ang II resulted in preservation of the EC phenotype. Compared to IL-6 and VEGF-A alone, the addition of Ang II increased the expression of VEGFR-2, Sox18, vWF, PECAM-1, and VE-cadherin (Figures 6(a)–6(e)). Ang II cotreatment also restored capillary tube formation (Figures 6(e) and 6(i)). Likewise, differentiation of naïve MSCs with the combination of TNF*α*, VEGF-A, and Ang II resulted in preservation of the EC phenotype (Figures 6(a)–6(f) and 6(i)).

The combination of IL-6, TNF*α*, and VEGF-A resulted in nearly complete inhibition of the expression of VEGFR-2, Sox18, vWF, PECAM-1, and VE-cadherin (Figures 6(a)–6(d)) and capillary tube formation (Figures 6(a)–6(c), 6(g) and 6(i)). In this case, the addition of Ang II failed to rescue the inhibitory effects of IL-6 plus TNF*α* on VEGF-A-mediated differentiation of MSCs into ECs (Figures 6(a)–6(c), 6(h), and 6(i)).

## 4. Discussion

Atherosclerosis is an inflammatory vascular disease, characterized by the infiltration of immune cells into plaques. Cardiovascular diseases are associated with a myriad of conditions and risk factors that promote systemic inflammation, including obesity, diabetes, high blood pressure, stress, smoking, and various viral and bacterial infections [1]. Coronary artery disease is a “silent killer” because the diagnosis of ischemic heart disease is frequently delayed until an acute event occurs. The right coronary artery and the left anterior descending artery are common sites of blockage [1, 2]. Symptoms of angina may arise from temporary loss of blood flow due to the spasm of a narrow coronary artery. Occlusion of coronary arteries can suddenly progress into a myocardial infarction upon rupture of an unstable atherosclerotic plaque [17]. The amount of time that the blockage impedes coronary blood flow determines the extent of damage to the heart muscle [1, 2].

Surgical intervention is required to restore coronary artery blood circulation. However, the surgical procedures also impose further complications and damage to the coronary arteries. Vascular injury initiates a cascade of events that lead to production of cytokines, chemokines, and growth factors by the local endothelium and smooth muscle layers [15–17]. Our group examined the local effects of arterial injury in previous studies using a swine model of coronary artery intimal hyperplasia. We found that increased levels of TNF*α* were associated with decreased vitamin D receptor and suppressor of cytokine signaling 3 within the neointimal region of coronary arteries after angioplasty [18, 19]. It is intuitive that such products are a mixture of both harmful and protective factors. Ultimately, the phenotype of vascular smooth muscle cells is modulated to increase their proliferation, migration, and synthetic activity, which contribute to repair a vessel after injury [22]. Endothelial production of VEGF-A may elicit a protective response to vascular injury. VEGF-A stimulates EC proliferation and the recruitment of peripheral stem cells [7–9].

Stem cell-based therapies have the potential to improve blood flow to the heart by either differentiating into vascular ECs directly or by supporting the differentiation of ECs, smooth muscle cells, and cardiomyocytes [3–6]. Human clinical trials using stem cell-based treatments have been performed in conjunction with balloon angioplasty and intravascular stenting [23, 24]. The main routes of transplanting stem cells are intracoronary and transendocardial delivery. The strategy of transplanting MSCs and differentiated ECs into the ischemic heart is intended to regenerate damaged vascular endothelium and to increase angiogenesis and vascularity. Thus far, clinical outcomes are largely inconclusive. The rates of reendothelialization are highly variable among patients [23–25]. Several factors released during the procedure could limit the process of reendothelialization following stem cell-based therapies, resulting in inadequate cell survival, engraftment, or differentiation. In the case of differentiated ECs, it is possible for the cells to lose their “EC-like” traits and functions within the transplant region.

In the present study, we assessed the impact of inflammatory cytokines on the molecular pathways that govern the differentiation of MSCs into ECs. VEGF-A has been characterized as a factor that coordinates the differentiation of MSCs into ECs* in vitro* [5, 9–11]. We found that the levels of IL-6, TNF*α*, and Ang II are increased at the PTCA interventional site within the LCX artery at long-term postinjury compared to control noninjured arteries. Next, we tested the effects of these inflammatory mediators on EC differentiation and function. IL-6 and TNF*α* inhibited VEGF-A-induced differentiation of MSCs into ECs and capillary tube formation. However, Ang II and VEGF-A produced a cooperative increase in markers of EC phenotype. Cotreatment with Ang II and VEGF-A effectively rescued EC marker induction and capillary tube formation despite the presence of IL-6 or TNF*α*. Effects on EC marker expression directly correlated with levels of VEGFR-2 and the transcription factor Sox18. These results suggest that angiotensin receptor signaling opposes receptor-response coupling pathways of IL-6 and TNF*α*.

**Figure 7:**
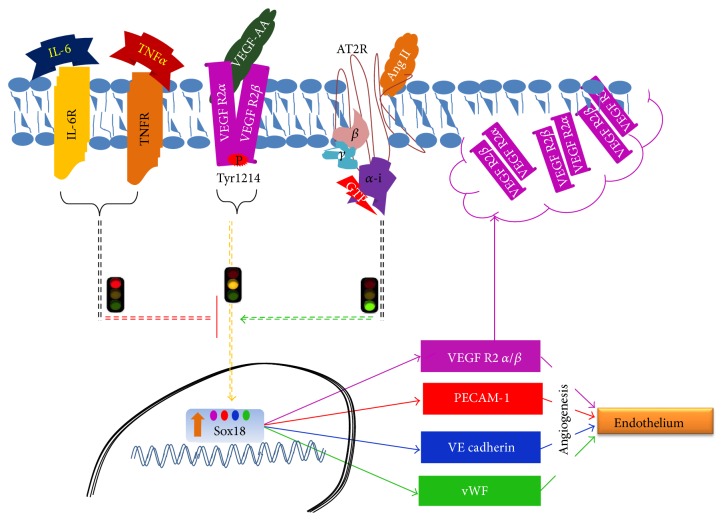
Schematic diagram showing interactions among receptors and signaling pathways during the differentiation of BM-MSCs into ECs.

In a recent study, our group demonstrated that Ang II promotes the differentiation of MSCs into functional ECs through an AT2R-dependent mechanism [20]. This is important because the proinflammatory actions of Ang II are mediated through AT1R signaling [26, 27]. Herein, we propose that local proinflammatory cytokine production causes failure of EC differentiation and angiogenesis near the sites of arterial occlusion and/or injury. The opposing signaling pathways are illustrated in Figure 7. These revelations could aid in the development of therapies geared towards replenishment of endogenous ECs as well as exogenous stem cell transplants in occlusive cardiovascular diseases.

In particular, AT2R-specific agonists, along with anti-inflammatory compounds (i.e., inhibitors of IL-6R and TNF*α*-R), are candidates for promoting angiogenesis and/or repair of damaged blood vessels. The use of angiotensin-converting enzyme inhibitors and AT1R receptor blockers is well established in the treatment of patients with cardiovascular disease [28–31]. Inhibition of angiotensin-converting enzyme has also been proposed as a means to interrupt the proangiogenic activity of Ang II [32]. In context with the new findings presented in this study, it may be possible to enhance the beneficial effects of existing treatments. Further preclinical and clinical testing is warranted to determine the combinatorial effects of the aforementioned agents in tandem with cell-based therapies in occlusive cardiovascular disease intervention.

## References

[1] Go A. S., Mozaffarian D., Roger V. L. (2013). Heart disease and stroke statistics—2013 update: a report from the American Heart Association. *Circulation*.

[2] Doostzadeh J., Clark L. N., Bezenek S., Pierson W., Sood P. R., Sudhir K. (2010). Recent progress in percutaneous coronary intervention: evolution of the drug-eluting stents, focus on the XIENCE v drug-eluting stent. *Coronary Artery Disease*.

[3] Mund J. A., Ingram D. A., Yoder M. C., Case J. (2009). Endothelial progenitor cells and cardiovascular cell-based therapies. *Cytotherapy*.

[4] Kuswardhani R. A. T., Soejitno A. (2011). Bone marrow-derived stem cells as an adjunctive treatment for acute myocardial infarction: a systematic review and meta-analysis. *Acta medica Indonesiana*.

[5] Pankajakshan D., Kansal V., Agrawal D. K. (2013). In vitro differentiation of bone marrow derived porcine mesenchymal stem cells to endothelial cells. *Journal of Tissue Engineering and Regenerative Medicine*.

[6] Burdon T. J., Paul A., Noiseux N., Prakash S., Shum-Tim D. (2011). Bone marrow stem cell derived paracrine factors for regenerative medicine: current perspectives and therapeutic potential. *Bone Marrow Research*.

[7] Coultas L., Chawengsaksophak K., Rossant J. (2005). Endothelial cells and VEGF in vascular development. *Nature*.

[8] Mayer H., Bertram H., Lindenmaier W., Korff T., Weber H., Weich H. (2005). Vascular endothelial growth factor (VEGF-A) expression in human mesenchymal stem cells: autocrine and paracrine role on osteoblastic and endothelial differentiation. *Journal of Cellular Biochemistry*.

[9] Neufeld G., Cohen T., Gengrinovitch S., Poltorak Z. (1999). Vascular endothelial growth factor (VEGF) and its receptors. *The FASEB Journal*.

[10] Xu J., Liu X., Jiang Y. (2008). MAPK/ERK signalling mediates VEGF-induced bone marrow stem cell differentiation into endothelial cell. *Journal of Cellular and Molecular Medicine*.

[11] Cai J., Jiang W. G., Ahmed A., Boulton M. (2006). Vascular endothelial growth factor-induced endothelial cell proliferation is regulated by interaction between VEGFR-2, SH-PTP1 and eNOS. *Microvascular Research*.

[12] Breuss J. M., Cejna M., Bergmeister H. (2002). Activation of nuclear factor-*κ*B significantly contributes to lumen loss in a rabbit lliac artery balloon angioplasty model. *Circulation*.

[13] Brull D. J., Montgomery H. E., Sanders J. (2001). Interleukin-6 gene -174G > C and -572G > C promoter polymorphisms are strong predictors of plasma interleukin-6 levels after coronary artery bypass surgery. *Arteriosclerosis, Thrombosis, and Vascular Biology*.

[14] Exner M., Schillinger M., Minar E. (2004). Interleukin-6 promoter genotype and restenosis after femoropopliteal balloon angioplasty: initial observations. *Radiology*.

[15] Tanaka H., Sukhova G., Schwartz D., Libby P. (1996). Proliferating arterial smooth muscle cells after balloon injury express TNF-*α* but not interleukin-1 or basic fibroblast growth factor. *Arteriosclerosis, Thrombosis, and Vascular Biology*.

[16] Viswanathan M., Strömberg C., Seltzer A., Saavedra J. M. (1992). Balloon angioplasty enhances the expression of angiotensin II AT1 receptors in neointima of rat aorta. *Journal of Clinical Investigation*.

[17] Kornowski R., Hong M. K., Tio F. O., Bramwell O., Wu H., Leon M. B. (1998). In-stent restenosis: contributions of inflammatory responses and arterial injury to neointimal hyperplasia. *Journal of the American College of Cardiology*.

[18] Gupta G. K., Agrawal T., Del Core M. G., Hunter W. J., Agrawal D. K. (2012). Decreased expression of vitamin D receptors in neointimal lesions following coronary artery angioplasty in atherosclerotic swine. *PLoS ONE*.

[19] Gupta G. K., Dhar K., Del Core M. G., Hunter W. J., Hatzoudis G. I., Agrawal D. K. (2011). Suppressor of cytokine signaling-3 and intimal hyperplasia in porcine coronary arteries following coronary intervention. *Experimental and Molecular Pathology*.

[20] Ikhapoh A. I., Pelham C. J., Agrawal D. K. (2015). Synergistic effect of angiotensin II on vascular endothelial growth factor-A-mediated differentiation of bone marrow-derived mesenchymal stem cells into endothelial cells. *Stem Cell Research & Therapy*.

[21] Ikhapoh I. A., Pelham C. J., Agrawal D. K. (2015). Sry-type HMG box 18 contributes to the differentiation of bone marrow-derived mesenchymal stem cells to endothelial cells. *Differentiation*.

[22] Owens G. K., Kumar M. S., Wamhoff B. R. (2004). Molecular regulation of vascular smooth muscle cell differentiation in development and disease. *Physiological Reviews*.

[23] George J. C. (2010). Stem cell therapy in acute myocardial infarction: a review of clinical trials. *Translational Research*.

[24] Hare J. M., Fishman J. E., Gerstenblith G. (2012). Comparison of allogeneic vs autologous bone marrow-derived mesenchymal stem cells delivered by transendocardial injection in patients with ischemic cardiomyopathy: the POSEIDON randomized trial. *Journal of the American Medical Association*.

[25] Forte A., Rinaldi B., Sodano L. (2012). Stem cell therapy for arterial restenosis: potential parameters contributing to the success of bone marrow-derived mesenchymal stromal cells. *Cardiovascular Drugs and Therapy*.

[26] Higuchi S., Ohtsu H., Suzuki H., Shirai H., Frank G. D., Eguchi S. (2007). Angiotensin II signal transduction through the AT1 receptor: novel insights into mechanisms and pathophysiology. *Clinical Science*.

[27] Carey R. M., Jin X.-H., Wang Z.-Q., Siragy H. M. (2000). Nitric oxide: a physiological mediator of the type 2 (AT2) angiotensin receptor. *Acta Physiologica Scandinavica*.

[28] Latini R., Maggioni A. P., Flather M., Sleight P., Tognoni G. (1995). ACE inhibitor use in patients with myocardial infarction: summary of evidence from clinical trials. *Circulation*.

[29] Demers C., Mody A., Teo K. K., McKelvie R. S. (2005). ACE inhibitors in heart failure: what more do we need to know?. *American Journal of Cardiovascular Drugs*.

[30] White H. D., Aylward P. E. G., Huang Z. (2005). Mortality and morbidity remain high despite captopril and/or valsartan therapy in elderly patients with left ventricular systolic dysfunction, heart failure, or both after acute myocardial infarction: results from the Valsartan in Acute Myocardial Infarction Trial (VALIANT). *Circulation*.

[31] Saunders E., Cable G., Neutel J. (2008). Predictors of blood pressure response to angiotensin receptor blocker/diuretic combination therapy: a secondary analysis of the irbesartan/hydrochlorothiazide blood pressure reductions in diverse patient populations (inclusive) study. *Journal of Clinical Hypertension*.

[32] Yoshiji H., Kuriyama S., Fukui H. (2002). Angiotensin-I-converting enzyme inhibitors may be an alternative anti-angiogenic strategy in the treatment of liver fibrosis and hepatocellular carcinoma: possible role of vascular endothelial growth factor. *Tumor Biology*.

